# PPAR*γ* and the Innate Immune System Mediate the Resolution of Inflammation

**DOI:** 10.1155/2015/549691

**Published:** 2015-12-02

**Authors:** Amanda Croasdell, Parker F. Duffney, Nina Kim, Shannon H. Lacy, Patricia J. Sime, Richard P. Phipps

**Affiliations:** ^1^Department of Environmental Medicine, University of Rochester School of Medicine and Dentistry, 601 Elmwood Avenue, Rochester, NY 14642, USA; ^2^Lung Biology and Disease Program, University of Rochester School of Medicine and Dentistry, 601 Elmwood Avenue, Rochester, NY 14642, USA; ^3^Department of Medicine, University of Rochester School of Medicine and Dentistry, 601 Elmwood Avenue, Rochester, NY 14642, USA

## Abstract

The resolution of inflammation is an active and dynamic process, mediated in large part by the innate immune system. Resolution represents not only an increase in anti-inflammatory actions, but also a paradigm shift in immune cell function to restore homeostasis. PPAR*γ*, a ligand activated transcription factor, has long been studied for its anti-inflammatory actions, but an emerging body of literature is investigating the role of PPAR*γ* and its ligands (including thiazolidinediones, prostaglandins, and oleanolic acids) in all phases of resolution. PPAR*γ* can shift production from pro- to anti-inflammatory mediators by neutrophils, platelets, and macrophages. PPAR*γ* and its ligands further modulate platelet and neutrophil function, decreasing trafficking, promoting neutrophil apoptosis, and preventing platelet-leukocyte interactions. PPAR*γ* alters macrophage trafficking, increases efferocytosis and phagocytosis, and promotes alternative M2 macrophage activation. There are also roles for this receptor in the adaptive immune response, particularly regarding B cells. These effects contribute towards the attenuation of multiple disease states, including COPD, colitis, Alzheimer's disease, and obesity in animal models. Finally, novel specialized proresolving mediators—eicosanoids with critical roles in resolution—may act through PPAR*γ* modulation to promote resolution, providing another exciting area of therapeutic potential for this receptor.

## 1. Introduction: Innate Immunity, Inflammation, and PPAR***γ***

**Figure 1:**
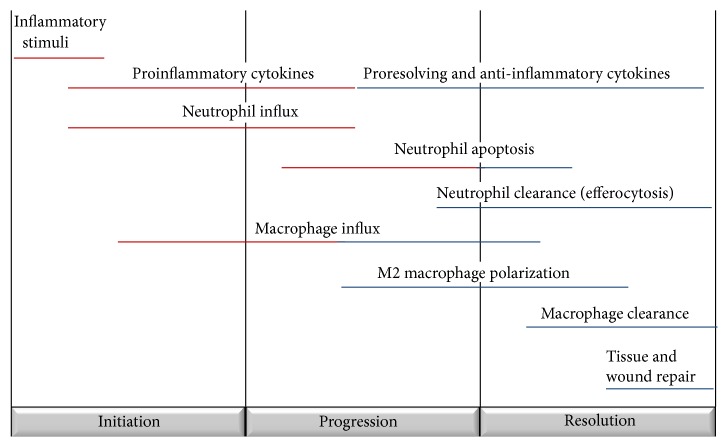
Inflammation and resolution are active and dynamic processes. The initiation, progression, and resolution of inflammation are characterized by unique cellular signals and trafficking.

Identification of the cardinal signs of inflammation (calor, dolor, rubor, and tumor) dates all the way back to the 1st century. Identifying the resolution phase of inflammation (long thought to be a passive process) is a far more recent advancement. We now know that resolution is an active and dynamic process and critical to the prevention of chronic and/or excessive inflammation [1]. Proresolving actions are distinct from anti-inflammatory processes; while anti-inflammatory molecules and medications act to dampen and suppress proinflammatory cells and signals, resolution represents a phenotypic shift in immune cell function towards repair and homeostasis. Resolution is characterized by several distinct phases. First, there is an end to the production of proinflammatory cytokines and halting of inflammatory neutrophil influx. Second, neutrophils present at the inflammatory site undergo apoptosis. Third, macrophages demonstrate a phenotypic switch and enhanced efferocytosis of apoptotic cells; the second and third phases coincide with increased production of anti-inflammatory and proresolving molecules. Finally, there is a clearance of macrophages, promotion of wound healing, and tissue repair to mediate the end of the inflammatory response [1]. These phases are frequently overlapping and the specific aspects of resolution can vary based on the inflammatory stimuli, organ location, and individual host characteristics (Figure 1).

Many aspects of the resolution of inflammation are mediated by the innate immune system. The innate immune system is comprised of a collection of cells, including neutrophils, macrophages, dendritic cells, eosinophils, basophils, platelets, and natural killer cells. These cells are responsible for recruiting other immune cells to sites of injury and infection, initiating complement cascades, activating the adaptive immune system, and removing foreign invaders and apoptotic cells. Neutrophils and macrophages are the first responders to inflammatory stimuli and are the first cells to begin to signal the resolution process. Furthermore, macrophages have in recent years been shown to be polarized towards multiple phenotypes, allowing this diverse cell type to contribute to both the proinflammatory and proresolving phases of inflammation [2].

**Figure 2:**
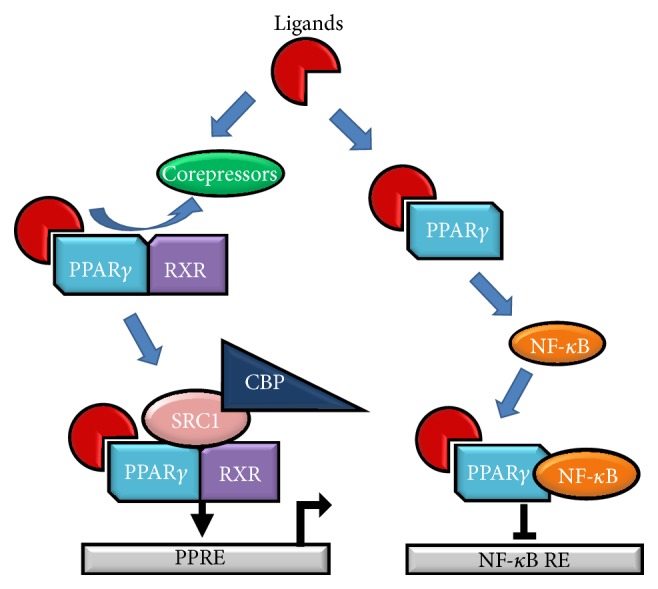
Overview of PPAR*γ* activation. PPAR*γ* typically exists as a heterodimer with RXR*α*, bound to corepressor molecules. Upon ligand stimulation, these corepressors are displaced and the ligand, PPAR*γ*, RXR*α*, and coactivators (such as CBP and SRC1) form an active complex, binding to PPAR*γ* response elements (PPRE). Alternatively, upon ligand stimulation PPAR*γ* alone can bind with NF-*κ*B to repress NF-*κ*B target genes.

Peroxisome proliferator activated receptors (PPARs) are a family of nuclear hormone receptors with three isoforms—PPAR*α*, PPAR*β*, and PPAR*γ*. PPAR*γ* has a wide variety of biological roles, including regulating fatty acid synthesis and storage and glucose metabolism, promoting adipogenesis, and inhibiting inflammatory signaling through NF-*κ*B. In recent years, the role of PPAR*γ* in mediating responses to inflammation has been of particular interest. PPAR*γ* is expressed on numerous immune cells, including monocytes/macrophages, platelets, lymphocytes, and dendritic cells [3–5]. PPAR*γ* usually exists as a heterodimer complexed with retinoid X receptor alpha (RXR*α*); these two molecules are typically bound to corepressors. Upon ligand stimulation, the corepressor molecules are displaced and ligand, PPAR*γ*, RXR*α*, and coactivators (such as CBP and SRC1) form an active complex, binding to PPAR*γ* response elements (PPRE). Alternatively, upon ligand stimulation PPAR*γ* can bind with NF-*κ*B to repress NF-*κ*B target genes (Figure 2). A wide variety of PPAR*γ* ligands have been identified which bind to and activate PPAR*γ* (Table 1). These ligands include thiazolidinediones (TZDs), which are antidiabetic drugs, as well as several prostaglandins (prostaglandin D_2_ (PGD_2_) and its metabolite, 15-deoxy prostaglandin J_2_ (15d-PGJ_2_)), oleanolic acids, and other eicosanoids. Importantly, ligand activation of PPAR*γ* has been shown to exert potent anti-inflammatory effects. In addition, several of these molecules have effects independent of PPAR*γ*, many of which are also anti-inflammatory. The degree to which these ligands act in an independent manner varies, with compounds such as ibuprofen acting predominantly independent of PPAR*γ*, prostaglandins exhibiting mixed dependent/independent activity, and TZDs acting in a heavily PPAR*γ*-dependent manner; the PPAR*γ*-independent effects of many of these ligands have been reviewed previously [6]. PPAR*γ* research has begun to focus on these anti-inflammatory effects and to investigate the role that PPAR*γ* and its ligands play in the resolution of inflammation.

Many of PPAR*γ*'s established anti-inflammatory effects have been shown to occur through innate immune signaling, particularly in monocytes and macrophages [7]. These cells are furthermore capable of producing a number of PPAR*γ* ligands, which can potentiate the anti-inflammatory and proresolving actions of this receptor on additional immune cells and other cells. Resolution is also mediated by a wide variety of signals, including cytokines and chemokines, apoptotic proteins, and eicosanoids. Eicosanoids, several of which are PPAR*γ* ligands, are produced through lipid class switching. For instance, under resolution there may be increased production of 15d-PGJ_2_ rather than proinflammatory prostaglandins, both of which come from arachidonic acid precursors [8]. Prostaglandins are produced under strong temporal regulation, with distinct shifts in which mediators are produced depending on the phase of inflammation. Furthermore, prostaglandin precursors can be broken down into additional prostaglandin isoforms; PGD_2_ is rapidly metabolized to 15d-PGJ_2_ [9]. While both of these mediators have anti-inflammatory effects, 15d-PGJ_2_ is a much more potent activator of PPAR*γ* [10]. Prostaglandins and other eicosanoids signal through multiple receptors to initiate resolution and appropriate cellular responses [11]. The roles of the PPAR*γ* transcription factor, and PPAR*γ* ligands, in resolution are beginning to be elucidated, and this receptor is emerging as an important player in all stages of the resolution of inflammation.

## 2. PPAR***γ*** and Altered Cytokine Production

PPAR*γ* has been shown in numerous studies to affect the expression of proinflammatory cytokines. In this review, we have focused on cytokines and chemokines that are particularly important for their pro- and anti-inflammatory effects on innate immune cells. First, PPAR*γ* has been shown to extensively affect expression of tumor necrosis factor-alpha (TNF*α*). TNF*α* is an important cytokine in regulating immune cell function and can act to induce fever, promote apoptosis, and stimulate other cytokines. TNF*α* can also act as a macrophage and neutrophil chemoattractant. While this cytokine has important roles in bacterial killing, excessive expression promotes chronic inflammation and poor health effects, such as rapid weight loss. In human neutrophils, TNF*α* actually increases mRNA and protein PPAR*γ* expression, likely in a compensatory mechanism or a feedback loop [12]. In conjunction with this increased expression, PPAR*γ* ligands, particularly TZDs, potently reduce TNF*α* expression [13]. Pioglitazone treated and lipopolysaccharide- (LPS-) exposed mice and guinea pigs have decreased TNF*α* expression. Indeed, both pioglitazone and rosiglitazone reduced TNF*α* expression in a wide range of inflammatory models, including sepsis, ischemia/reperfusion, colitis, gastric injury, and spinal trauma models [14–19]. These effects were independent of the route of administration, and the oral delivery of pioglitazone to decrease TNF*α* in mouse livers is particularly interesting from a therapeutic standpoint [20]. These effects were largely shown to be PPAR*γ*-dependent, with the reduction of TNF*α* expression blocked by PPAR*γ* antagonists [17, 19]. Other PPAR*γ* ligands are also capable of dampening TNF*α* expression, including 15d-PGJ_2_ and oleanolic acid [21–23].

Along with TNF*α*, several interleukins (ILs) are produced in response to inflammatory stimuli. IL-6 is a component of the acute inflammatory response and a mediator of fever. Pioglitazone, rosiglitazone, and ciglitazone decrease local production of IL-6 in the intestine; colon; and lung, colon, and liver, respectively [14, 18, 22]. 15d-PGJ_2_ and PGD_2_ microspheres similarly reduced bacteria stimulated increases in IL-6 [22, 24]. IL-8 is a key chemokine for neutrophil trafficking. IL-8 is broadly expressed by a multitude of cell types, including macrophages, and induced by a variety of inflammatory stimuli. Rosiglitazone prevents tobacco smoke induced decreases in PPAR*γ*, induction of leukotrienes, and IL-8 production [25]. IL-1*β* is also produced to promote acute inflammation and is reduced by rosiglitazone, 15d-PGJ_2_, and pioglitazone in a PPAR*γ*-dependent manner [15, 17, 26]. The broad ranging effects of PPAR*γ* ligands on proinflammatory cytokines may be due to PPAR*γ* effects on the NF-*κ*B pathway, as PPAR*γ* has been shown to decrease NF-*κ*B expression [16–18, 27].

Finally, PPAR*γ* is also involved in the production of anti-inflammatory and proresolving cytokines, though these data remain controversial. IL-10, for instance, is a cytokine produced under both pro- and anti-inflammatory conditions. In the context of resolution, IL-10 can be produced by macrophages to mediate proresolving effects. Rosiglitazone induces IL-10 production in experimental colitis and Parkinson's models of disease [18, 26], but in a septic lung 15d-PGJ_2_ and pioglitazone reduced IL-10 expression [18, 22]. Whether these differences are due to differences in inflammatory stimuli, PPAR*γ* ligand specific signaling, the time points chosen, or observed organ system requires additional studies to conclusively answer. Rosiglitazone similarly induced TGF*β* expression, another proresolving cytokine, in microglia in Parkinson's model of disease [26]. This shift in cytokine production, rather than suppression of all cytokine signaling, implicates that PPAR*γ* is simply acting in not only an anti-inflammatory manner, but a proresolving one as well.

## 3. PPAR***γ*** and Neutrophil Apoptosis and Clearance

Neutrophils are the first responders to most inflammatory stimuli. These cells are rapidly produced, with quick influx to a site of injury. Upon arrival at the injured site, they are responsible for phagocytosis of foreign invaders and cytokine/chemokine production to recruit other immune cells. An excessive influx of neutrophils, failure to shut down cellular influx, and a lack of clearance can lead to neutrophilia, a hallmark of many inflammatory diseases. Appropriate neutrophil apoptosis and clearance, as well as prevention of too many neutrophils trafficking to an inflammatory site, are a key component of resolution. PPAR*γ* promotes apoptosis of a variety of cell types, and knockdown of PPAR*γ* strongly decreases uptake of apoptotic cells [28]. Our focus here will be on the influx, apoptosis, and clearance of neutrophils.

The effect of one TZD, pioglitazone, on neutrophil functions has been particularly well characterized. Pioglitazone treated mice and guinea pigs have decreased neutrophil numbers and myeloperoxidase (MPO) expression in an LPS model; MPO is frequently used as a marker of neutrophil presence [13, 29]. Other models of inflammation yielded similar effects, with pioglitazone decreasing MPO activity in mice on a high-fat diet and mice with bacterial sepsis [14, 20]. Pioglitazone also decreased neutrophil counts specifically in a mouse model of ischemia/reperfusion. Interestingly, there is less apoptosis in mouse hepatic cells in this model, suggesting either specific regulation of cellular apoptosis, wherein neutrophils are targeted but other cell types are impervious to PPAR*γ* induced apoptosis, or a prevention of neutrophil influx in the first place. PPAR*γ* expression increased immediately following injury, which could support a prevention of influx at this early time point [15]. Similar results were seen in a spinal trauma model, where another TZD, rosiglitazone, both increased PPAR*γ* expression and prevented neural cell apoptosis. These effects were all prevented by GW9662, a PPAR*γ* antagonist, thus implying a PPAR*γ*-dependent mechanism [19]. Since rosiglitazone has been shown in other studies of colitis and gastric injury to reduce MPO activity [16–18, 27], TZDs could be acting to specifically target neutrophil apoptosis, though the exact mechanisms of this remain unclear.

Prostaglandins also play a highly important role in neutrophil trafficking and apoptosis. For example, 15d-PGJ_2_ both reduces MPO activity and directly reduces neutrophil numbers [30, 31]. However, 15d-PGJ_2_ is produced by alveolar macrophages before neutrophil infiltration, which could be an early feedback mechanism rather than induction of neutrophil apoptosis [32]. 15d-PGJ_2_ decreases in MPO activity and beneficial effects were associated with increased PPAR*γ* DNA-binding, though a PPAR*γ* antagonist did not block these effects, indicating they may be at least partially PPAR*γ*-independent [22]. In 15d-PGJ_2_-treated mice with induced spinal cord injuries, the spinal cord MPO activity was significantly attenuated in comparison to vehicle treated mice. In this experiment, coadministration of GW9662 and 15d-PGJ_2_ significantly blocked the effect of the PPAR*γ* agonist on neutrophil infiltration. There was an observed decrease in the number of apoptotic bodies, but this was not specifically tied to neutrophils [23]. The strongest evidence for 15d-PGJ_2_ induced neutrophil apoptosis is demonstrated by Gilroy and colleagues using a rat model of pleurisy. Cyclooxygenase-2 (Cox-2) was originally shown to promote neutrophil apoptosis and resolution, correlating with increases in PGD_2_ and 15d-PGJ_2_. Indomethacin (a nonspecific Cox inhibitor) and specific Cox-2 inhibitors prevented induction of neutrophil apoptosis, but addition of 15d-PGJ_2_ along with these inhibitors increases both neutrophil and macrophage apoptosis, showing specific actions of this prostaglandin [33].

PGD_2_ more clearly contributes to neutrophil trafficking and apoptosis, as increased PGD_2_ levels correspond to spontaneous neutrophil apoptosis. Incubating human neutrophils with PGD_2_ and 15d-PGJ_2_ also induced apoptosis in a dose-dependent manner. Furthermore, human macrophages incubated with opsonized and nonopsonized apoptotic neutrophils produce increased levels of PGD_2_. This correlates with clinical data, as certain patients fail to produce PGD_2_ in response to apoptotic neutrophils and have increased neutrophilia [34]. MPO is increased in correlation with PGD_2_ decreases in colonic inflammation, and exogenous addition of PGD_2_ decreases MPO activity and infiltration of neutrophils. While PPAR*γ* expression does increase in this inflammatory model, whether or not PGD_2_'s effects were PPAR*γ*-independent or PPAR*γ*-dependent was not evaluated [35]. There are clearly complex mechanisms at play regarding the PPAR*γ*-independent and PPAR*γ*-dependent effects of prostaglandins, but the overall evidence suggests that these mediators contribute to neutrophil influx, clearance, and apoptosis and that these regulatory pathways merit further investigation.

Several studies have examined both TZDs and PGs side-by-side. 15d-PGJ_2_ and pioglitazone both reduced neutrophil numbers and MPO activity in a caecal-ligation puncture model of sepsis. Importantly, while most studies see local effects of PPAR*γ* ligands, this study showed decreased MPO activity in the lung, liver, and colon. These two ligands were able to rescue sepsis-induced decreases in PPAR*γ* expression in the lung to normal constitutive levels [22]. In another study, treatment with either 15d-PGJ_2_ or troglitazone decreased human neutrophil chemotaxis in response to IL-8 but did not induce apoptosis. These results were PPAR*γ*-dependent, as direct transfection with a constitutively active PPAR*γ* gene also inhibited chemotaxis. These two ligands do appear to have unique pathways, though, as 15d-PGJ_2_ abolished ERK1/2 phosphorylation, while troglitazone dampened ERK-P but also inhibited neutrophil actin polymerization. This may not always be a beneficial response, as sepsis patients have higher expression of PPAR*γ*, and blocking PPAR*γ* reverses sepsis-induced inhibition of neutrophil chemotaxis [12].

Along with PGs and TZDs, oleanolic acid (OA) decreased neutrophils in bronchoalveolar lavage and decreased apoptosis of all cells across all other organ systems. Since this antiapoptotic effect was seen in all cell types, including neutrophils, OA is likely acting to prevent neutrophil influx [36]. Oleanolic acid-NO_2_, though, also decreased neutrophil counts in allergic airway disease and this reduction was due to induction of neutrophil apoptosis. OA-NO_2_ stimulated PPAR*γ* binding and nuclear translocation, and differences in receptor binding may account for these different observed effects [21]. Another PPAR*γ* ligand, CDDO, additionally decreased neutrophil numbers and MPO activity [37, 38].

There is additional evidence that neutrophils have an anti-inflammatory role and contribute to the resolution of inflammation. In a stroke model, rosiglitazone increased the numbers of neutrophils in the brain, and the protective effects of rosiglitazone were abolished after neutrophil depletion. These neutrophils express markers characteristic of M2 macrophages and are increased by rosiglitazone treatment. These “N2” neutrophils are preferentially phagocytosed by microglia [39]. While more extensive research is needed to fully characterize this potential new cell phenotype, the existence of multiple activation states for neutrophils is an interesting development in resolution biology, as is the role for PPAR*γ* in modulating these cells.

## 4. PPAR***γ*** and Macrophage Trafficking

Monocytes and macrophages traffic to sites of injury in both the inflammatory and resolution phases of inflammation. Currently, studies regarding the effects of PPAR*γ* and PPAR*γ* ligands on monocyte trafficking are contradictory and complex. Multiple TZDs, including troglitazone and pioglitazone, have been shown to increase expression of monocyte chemoattractant protein-1 (MCP-1) and bone marrow monocyte/macrophage recruitment in the rat kidney, but these same TZDs suppressed expression of MCP-1 and the MCP-1 receptor CCR2, inhibited migration in human cells, and reduced macrophage counts in other mouse models [40–44]. When PPAR*γ* is specifically knocked out in colon macrophages, mice had higher levels of CCR2 and MCP-1 [45].

These variable effects may be dependent on the inflammatory stimuli. Ciglitazone, for instance, dose-dependently inhibited monocyte chemotaxis due to plasmin stimulation, but not* N*-formyl-met-leu-phe- (FMLP-) induced influx, and may specifically target plasmin-induced chemotaxis pathways [46]. Rosiglitazone was further shown to decrease expression of CCR2 in a PPAR*γ*-dependent manner in primary human blood monocytes in the absence of any inflammatory stimuli but did not further decrease expression levels after 48 hours [47]. PPAR*γ* directly regulates CCR2 expression in mouse cells, leading to similar early decreases [48]. Taken together, these data imply that TZDs may be acting to “quiet” monocytes, preventing an early influx, but allowing alternative macrophage recruitment in the later stages of resolution to allow for cell clearance and tissue repair. Future studies should pay particular attention to PPAR*γ* and its effects on the temporal regulation of macrophage chemotaxis.

In contrast to the complicated literature regarding TZDs, prostaglandins much more clearly decrease macrophage influx. 15d-PGJ_2_ inhibited macrophage chemotaxis towards zymosan-treated serum and specifically inhibited macrophage trafficking to the site of damage in chronic liver injury (T cell, dendritic cell, and neutrophil trafficking were not affected) [49, 50]. While 15d-PGJ_2_ does not affect MCP-1 expression, it decreases CCR2 expression and inhibits migration of monocytes/macrophages [40, 41]. Mice deficient in PGD_2_ synthase, and thereby deficient in PGD_2_ and 15d-PGJ_2_, have increased MCP-1 and increased macrophage accumulation. This was due in part to a lack of D and J series prostaglandins which would normally act to prevent macrophage influx, but also largely due to impaired clearance of leukocytes from draining lymphatics, indicating a role for prostaglandins in the end stage clearance of macrophages [31]. In contrast to the abilities of TZDs to enhance early monocyte/macrophage chemotaxis, prostaglandins act later in the inflammatory process to prevent excessive inflammatory monocyte/macrophage influx and to clear cells at the end of resolution.

Discrepancies in PPAR*γ* ligand induced macrophage recruitment may also be due to differences in macrophage populations. PPAR*γ* is expressed highly in lung and spleen macrophages but has very low expression in peritoneal macrophages. One particular study additionally showed that inflammatory monocytes recruited to the site of inflammation expressed increased levels of PPAR*γ* as they differentiated to macrophages. PPAR*γ* deficiency in these infiltrating monocytes did not impair the initiation of inflammation but inhibited the resolution of inflammation, with increased neutrophils and inflammatory cytokine production in macrophage specific PPAR*γ* knockout mice. Furthermore, PPAR*γ* deletion in lung macrophages, but not spleen macrophages, impaired resolution, though macrophage numbers were consistent in all groups [51]. These data would suggest that the effect of PPAR*γ* monocyte and macrophage trafficking is secondary and that the more potent PPAR*γ*-dependent actions occur through macrophages already present at the site of inflammation.

## 5. PPAR***γ*** Enhances Macrophage Phagocytosis

A key aspect of resolution is enhanced macrophage phagocytosis of neutrophils, bacterial components, and other cell debris. PPAR*γ* and its agonists have been shown to reduce macrophage inflammatory activation while enhancing phagocytosis [7]. PPAR*γ* is increased in macrophages in the presence of apoptotic cells and can act on expression of multiple proteins linked to phagocytosis [52]. For instance, PPAR*γ* expression is necessary for basal expression of CD36, a major scavenger receptor [53, 54]. Induced increases in PPAR*γ*-RXR expression by multiple agonists also increased expression of CD36 and phagocytosis of* Plasmodium falciparum*-parasitized erythrocytes. These effects were seen in both the human monocytic THP-1 cell line and primary human blood monocytes [55]. PPAR*γ* may also act through IL-13 to enhance phagocytosis, since PPAR*γ* is necessary for IL-13 induced production of 15d-PGJ_2_, alternative activation of macrophages, and enhanced phagocytosis of parasitized erythrocytes [54]. PPAR*γ* is also involved in IL-13 induction of Dectin-1, which is required for* Candida albicans* clearance and resolution of yeast-induced inflammation [56].

Thiazolidinediones are the most potent group of PPAR*γ* ligands for enhancing macrophage phagocytosis. In brain abscesses in C57BL/6 mice, ciglitazone dose-dependently decreased bacterial load, with greater decreases seen after multiple days of postinfection treatment. Primary microglia were further isolated and treated with ciglitazone; ciglitazone increased microbial uptake of* Staphylococcus aureus*. Additionally, the edge of the abscess areas showed enhanced PPAR*γ* activity, demonstrating the contribution of PPAR*γ* to this decreased bacterial burden [57]. Similarly, mice given intraperitoneal doses of ciglitazone following intranasal* S. pneumonia* infections had decreased bacterial burdens. In contrast to the brain, though, mouse alveolar macrophages treated* in vitro* with ciglitazone had no alteration of phagocytosis, demonstrating a difference in ligand-responsiveness between macrophage types [58]. Rosiglitazone also enhanced phagocytosis by macrophages. Peripheral blood monocytes had increased CD36 expression and enhanced uptake of* P. falciparum* following rosiglitazone treatment. These effects were mirrored in a mouse model of cerebral malaria. Mice that received rosiglitazone infused chow had reduced parasite levels, dependent on CD36 expression. This treatment was effective as late as 5 days after infection [59]. In other models of neural inflammation, rosiglitazone again induced expression of CD36, which enhanced phagocytosis of neutrophils and promoted resolution; these effects were blocked by PPAR*γ* antagonists or PPAR*γ* gene knockdown [60, 61]. Other TZDs including pioglitazone and troglitazone similarly increased macrophage or microglial phagocytosis and CD36 expression in a PPAR*γ*-dependent manner [62, 63]. These phagocytic enhancements appear to be dependent on macrophage tissue origin. In one study, troglitazone and rosiglitazone were shown to enhance Fc*γ* receptor mediated phagocytosis of both beads and opsonized bacteria, but these increases were only seen in alveolar macrophages and not peritoneal macrophages [64]. These differences may be due to a difference in PPAR*γ* expression between different macrophage types and responsiveness to specific TZD ligands [51].

Conjugated linoleic acids (CLAs) also play a role in modulating phagocytosis. RAW264.7 cells (a mouse macrophage cell line) treated with CLAs had a dose-dependent increase in uptake of latex beads which was attenuated by the PPAR*γ* antagonist GW9662 [65]. Additionally, human monocytes incubated with CLAs and differentiated to macrophages had enhanced phagocytosis [66]. Another lipid molecule, OA-NO_2_, demonstrated similar actions and increased CD36 and macrophage phagocytosis [21]. While only a few studies exist examining the role of CLAs and OAs on phagocytosis, these data are promising, and low density lipoproteins and triterpenoids represent two under examined classes of PPAR*γ* ligands that may have potent proresolving effects.

In contrast to other PPAR*γ* ligands, PGD_2_ and 15d-PGJ_2_ both decrease macrophage phagocytic abilities. Human primary blood monocytes directly treated with PGD_2_ had mild dose-dependent inhibition of apoptotic neutrophil uptake [67]. While a study using PGD_2_ microspheres showed that these spheres were phagocytosed more efficiently than unloaded spheres, the PGD_2_ spheres also activated NF-*κ*B inflammatory pathways to a greater extent, indicating that this enhanced uptake promotes inflammatory pathways and not resolution phagocytosis [24]. Similar to PGD_2_, 15d-PGJ_2_ inhibited phagocytosis of* E. coli* in isolated macrophages [49]. 15d-PGJ_2_ also dampened phagocytosis of* Salmonella enterica* by mouse macrophages, and infected macrophages actually produced higher levels of 15d-PGJ_2_ and other prostaglandins in a feed-forward loop [68]. 15d-PGJ_2_ further inhibited macrophage phagocytosis of latex beads by both mouse bone marrow derived macrophages and the mouse RAW264.7 cell line [50, 69]. There may be a temporal aspect to these studies that has not yet been conclusively evaluated, as prostaglandins are known to be produced under strong temporal regulation. Particularly, it appears that prostaglandins play a larger anti-inflammatory role early in the resolution process rather than acting to enhance macrophage functions.

PPAR*γ* ligand effects on phagocytosis appear to be broad ranging, as PPAR*γ* ligands are able to enhance uptake of both opsonized and nonopsonized targets [64] and a broad range of pathogens including parasites and bacteria. These effects largely seem to be independent of the route of administration of ligands, as oral, intraperitoneal, and intravenous treatments all have demonstrated efficacy. Critical to further understanding of the ability of PPAR*γ* ligands to enhance phagocytosis is evaluating their dependent and independent actions, since many of these studies have not yet addressed these questions.

## 6. PPAR***γ*** and Alternative Macrophage Activation

Recent studies have begun to distinguish two broad classes of macrophages: classically activated (M1) and alternatively activated (M2). Classically activated macrophages are associated with a proinflammatory phenotype. These cells are activated by a number of traditional inflammatory stimuli, including IFN*γ* and LPS. M1 macrophages produce numerous proinflammatory cytokines and chemokines, have increased levels of iNOS, and demonstrate enhanced abilities to kill intracellular pathogens. M2 macrophages are characterized by their roles in the resolution phase of inflammation and in tissue repair and are stimulated by IL-4, IL-13, IL-10, and TGF*β*.

PPAR*γ* is the principal, but not exclusive, member of the PPAR family in promoting M2 macrophages [70–72]. PPAR*γ* response elements were identified in the promoter region of Arg-1, a key M2 marker, and confirmed by an electrophoretic mobility shift assay. Arg-1 expression is significantly decreased in macrophage specific PPAR*γ* knockout mice. Further supporting the link between PPAR*γ* and Arg-1, PPAR*γ* activates an Arg-1 luciferase assay, and this activation is further potentiated by the addition of rosiglitazone [73].

PPAR*γ* ligands play a particularly strong role in M2 polarization in the neural system. Pioglitazone promoted a shift from M1 to M2 macrophages and enhanced A*β* amyloid phagocytosis in the brain [74]. Rosiglitazone similarly induced CD206 expression (an M2 marker) in microglia in Parkinson's model of disease [26]. Along with modulating neuronal inflammation, TZDs can modulate pain responses through M1/M2 polarization. Local administration of rosiglitazone induced IL-10 production and M2 macrophage polarization and attenuated pain sensitivity responses. These effects were shown to be macrophage mediated [75, 76].

TZDs also promote M2 macrophages in other disease states. Pioglitazone enhanced M2 polarization in atherosclerotic lesions, diet-induced obesity, and a model of insulin resistance [77–79]. Rosiglitazone likewise enhanced expression of Arg-1 and CD206 in peritoneal and adipose tissue macrophages and in an* in vitro* model of COPD [80–82]. While rosiglitazone prevents specific M2c polarization (as defined by CD163 expression), troglitazone induced CD163 expression and dampened CD80 (M1) expression in human macrophages derived from blood monocytes [83, 84]. This wide range of actions is encouraging and points to common mechanisms of action and broader therapeutic use of these drugs. In general, TZDs appear to be the most potent PPAR*γ* ligands regarding the induction of M2 polarization.

In contrast to the TZDs, oleanolic acid (OA) inhibited M2 polarization and IL-10 secretion from human macrophages. There is some evidence that M2 macrophages polarized under certain stimuli are associated with tumor growth and/or cancer; thus OA may be acting to prevent tumor-associated macrophage production [85]. Oxidized LDL also accumulated in M2 macrophages and enhanced their proinflammatory capabilities, generating a cell type with M2 markers but higher production of proinflammatory cytokines [86], but was shown in a different study to promote a traditional M2 phenotype with decreased inflammatory cytokine production in THP-1 cells [87]. In contrast, CLA promoted increased IL-10 and M2 polarization of bone marrow derived macrophages atherosclerotic lesions, suggesting that all fatty acids do not have the same PPAR*γ*-dependent modulatory effects or that these ligands are acting in distinct PPAR*γ*-independent manners [88].

Prostaglandins also have unique roles in promoting M2 macrophages. While PGD_2_ and 15d-PGJ_2_ have so far been shown to decrease phagocytic abilities in macrophages, they have both been shown to promote M2 polarization. Mouse peritoneal macrophages treated with 15d-PGJ_2_ had potent increases in numerous M2 markers, including Arg-1, CD206, and TGF*β* [72]. PGD_2_ was also shown to promote an M2 phenotype, with increased levels of Arg-1 and CD206, though TNF*α* was also increased. Since PGD_2_ synthase is elevated in the differentiation of monocytes to macrophages, there may be mix of pro- and anti-inflammatory targets for this molecule, though it has been shown to be produced by macrophages to act in an autologous manner and promote M2 polarization [89]. Since M2 macrophages can be divided into further subclasses and mixed M1/M2 macrophage populations exist, further investigations are needed to fully elucidate the effects of prostaglandins on M2 phenotypes and functions.

Multiple other signals can also enhance PPAR*γ* expression and thereby M2 signaling. For example, deficiency in Cx3Cr1 led to increased PPAR*γ* expression on macrophages along with increased Arg-1 and decreased iNOS [90]. Netrin, adiponectin, and even exercise increased PPAR*γ* expression and an array of M2 genes [91–94]. The effects of PPAR*γ* may also be dependent on the type of macrophage assessed, as human alveolar macrophages have much higher PPAR*γ* expression than blood monocytes and are thought to be more predisposed towards an M2 state [3].

Importantly, this PPAR*γ* mediated induction of M2 macrophages plays a critical role in tissue repair and regeneration. These properties are highlighted in two diverse models, corneal scarring and diabetic wound healing. Alkali burn models are often used to induce optical wounds and scarring in mice, and PPAR*γ* expression is increased following alkali injury. Overexpression of PPAR*γ* in mouse corneal macrophages led to reduced proinflammatory cytokine and matrix metalloproteinase expression. These macrophages, in conjunction with fibroblasts and epithelial cells, contributed to reduced corneal scarring and faster reepithelialization and wound healing [95]. Similar effects were seen with administration of an ophthalmic pioglitazone solution, wherein treated mice had fewer myofibroblasts, decreased proinflammatory cytokine production, and more infiltrating M2 macrophages in the cornea compared to vehicle treated mice [96]. The inhibitory actions of PPAR*γ* and PPAR*γ* ligands (including TZDs and 15d-PGJ_2_) were observed in other optical scarring models, including TGF*β*-induced scarring and scratch wounds [97, 98].

**Figure 3:**
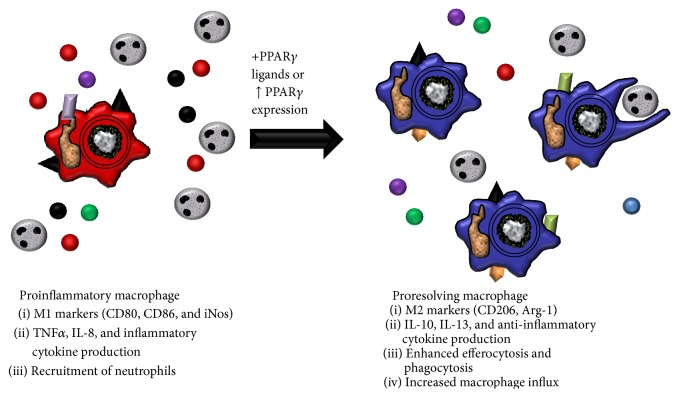
PPAR*γ* and macrophage activation. Proinflammatory macrophages are characterized by M1 activation markers, production of proinflammatory cytokines, and increased recruitment of immune cells. Upon stimulation with PPAR*γ* ligands and/or increases in PPAR*γ* expression, macrophages shift to an alternative M2 phenotype, with decreased proinflammatory actions, increased efferocytosis and phagocytosis, and production of anti-inflammatory cytokines.

Along with optical scarring, M2 macrophages contribute to tissue repair in diabetics. In a study by Mirza and colleagues, diabetic mice were subjected to excisional wounding [99]. Diabetic mice had impaired wound healing which correlated with decreased PPAR*γ* expression in mouse macrophages. Furthermore, loss of PPAR*γ* from wild type macrophages led to delayed reepithelialization and impaired wound closure. 15d-PGJ_2_ and rosiglitazone both acted to reverse the diabetic proinflammatory environment and to promote wound healing [99]. The beneficial actions of PPAR*γ* and its ligands in diabetic repair were modulated by M2 macrophage induction [73, 99]. These two disease states, along with multiple others, highlight the contribution of PPAR*γ* and M2 macrophages to tissue repair and wound healing. In summary, PPAR*γ* acts on multiple aspects of macrophages to mediate their key roles in resolution (Figure 3).

## 7. PPAR***γ*** and Platelets

Platelets are anucleate cells in the innate immune system with important roles in hemostasis and inflammation. Platelets are produced by megakaryocytes and in turn produce microparticles; all three of these cells or cell components can modulate the function of other immune cells and release cytokines. Meg-01 cells (a human megakaryocyte cell line), primary human megakaryocytes, and primary human platelets were all shown by our lab to express PPAR*γ* protein, though platelets did not contain PPAR*γ* mRNA [4]. Platelets can package PPAR*γ* in microparticles, which can then be taken up by leukocytes and other recipient cells [100]. PPAR*γ* released in these microparticles retains DNA-binding ability and can thus alter target cell functionality [100]. In a study from our lab using microparticles engineered to express high levels of PPAR*γ*, monocyte recipient cells had decreased production of proinflammatory mediators and increased CD36 expression following PPAR*γ*-microparticle uptake [101]. PPAR*γ* ligands are also capable of increasing platelet production, demonstrating a possible feed-forward loop for this receptor [102].

Platelets are capable of producing prothrombotic mediators that can alter the function of other immune cells. Plasminogen activator inhibitor 1 (PAI-1) is a serine proteinase inhibitor with multiple roles, including enhancement of leukocyte trafficking and recruitment. Patients given pioglitazone, troglitazone, or rosiglitazone in separate studies had significantly lower levels of PAI-1 in their serum [103–105]. TZDs have also been used therapeutically to reduce C-reactive protein (CRP), an acute-phase protein that increases following IL-6 secretion. These studies showed that patients who were given rosiglitazone or pioglitazone for 12–26 weeks have decreased serum levels of CRP and, where evaluated, IL-6 [106–109]. Troglitazone was further able to dampen thromboxane B2, a metabolite of prothrombotic thromboxane A2, in platelet-like human erythroleukemia cells and in human platelets [110].

**Figure 4:**
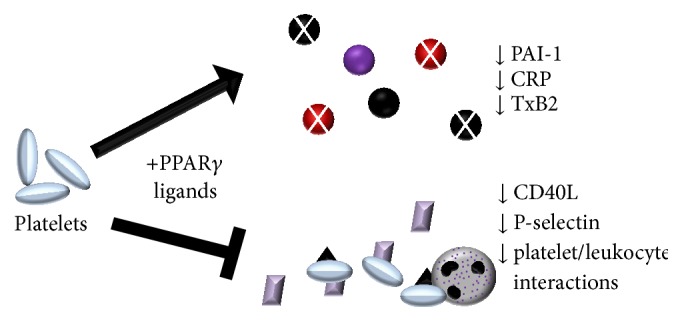
PPAR*γ* and platelet function. Upon stimulation with PPAR*γ* ligands, platelets decrease expression of multiple proinflammatory proteins and lipids, including PAI-1, CRP, and TxB2. PPAR*γ* ligands also decrease expression of P-selectin and CD40L, thereby decreasing the number of proinflammatory platelet/leukocyte aggregates.

Platelets can furthermore affect neutrophil trafficking by altering cellular adhesion and platelet/leukocyte interactions. Two particular molecules are key for mediating these effects, P-selectin and CD40L, both of which are highly expressed by platelets. P-selectin upregulation by platelets leads to enhanced chemokine synthesis and tethering of leukocytes in inflammatory sites, along with activation of NF-*κ*B [111]. Diabetic mice treated with pioglitazone had lower levels of soluble and platelet P-selectin [112]. Complementing these results, rosiglitazone decreased the percentage of P-selectin expressing platelets in human diabetic patients [106]. Activated platelets are also the most important source of soluble CD40L, which binds to CD40 to mediate platelet/leukocyte interactions [113]. Furthermore, platelets from CD40L knockout mice fail to form these platelet/leukocyte aggregates and have decreased MCP-1 levels [113]. Diabetic patients treated with rosiglitazone also had decreased levels of circulating CD40L after 12 weeks or three months of treatment [114, 115]. Direct treatment of human platelets with 15d-PGJ_2_ or rosiglitazone also decreased levels of surface CD40L and soluble CD40L [4, 116]. The PPAR*γ* antagonist GW9662 prevented these decreases, highlighting that these effects are PPAR*γ*-dependent [116]. These results underscore that the function of platelets as immune cells is a crucial, yet understudied, area of research, particularly given the clear importance these cells have in modulating leukocyte function during inflammation and resolution (Figure 4).

## 8. PPAR***γ*** and the Adaptive Immune Response

Along with the innate immune system, the adaptive immune system plays many roles in the resolution of inflammation. The effects of PPAR*γ* on T cells have been reviewed elsewhere, and there is emerging evidence for the effects of PPAR*γ* on B cells, which are known to express PPAR*γ* [5]. Initial studies investigated the role of endogenous PPAR*γ* agonists such as 15d-PGJ_2_ and synthetic ligands on B cell development and activation. High concentrations of PPAR*γ* ligands (*μ*M range) inhibit B cell proliferation and induce apoptosis in both normal and malignant B cells [5]. B cells treated with PPAR*γ* ligands demonstrated characteristic markers of apoptosis, including reduction in mitochondria membrane potential, activation of caspases 3 and 9, and consequently increase in the cleavage of the substrate PARP [117, 118]. The ability of PPAR*γ* agonists to induce apoptosis and suppress proliferation resulted from inhibition of NF-*κ*B, thereby blocking the transcription of downstream prosurvival mediators [118]. PPAR*γ* ligands can also directly prevent the activity of IKK, reducing the activation of NF-*κ*B [119]. Another study has shown that MAPKs are involved in mediating the effects on PPAR*γ* ligands to induce apoptosis in a mouse bone marrow pro-B cell line, indicating multiple mechanistic target pathways that may induce B cell apoptosis [120].

B lymphoma cells such as Burkitt lymphoma, Hodgkin and non-Hodgkin B cell lymphomas are known to have constitutively activated NF-*κ*B which can lead to enhanced B cell proliferation and survival [119]. In this context, PPAR*γ* agonists could be potential anticancer therapeutics in those tumors where NF-*κ*B appears to play a unique survival role. However, many PPAR*γ* agonists, especially 15d-PGJ_2_, have both PPAR*γ*-dependent and PPAR*γ*-independent effects which could be mediating the proresolving actions seen here [118]. Our lab used the Ramos B cell lymphoma cell line transfected with a dominant negative (DN) PPAR*γ* construct to block the activity of endogenous PPAR*γ* [121]. Surprisingly, 15d-PGJ_2_ maintained its antiproliferative effects on B cells regardless of the presence of DN PPAR*γ* construct, suggesting PPAR*γ*-independent actions of 15d-PGJ_2_ in lymphoma cells. The PPAR*γ*-independent effects included enhanced ROS production and reduced glutathione-S levels, which in turn lead to cell apoptosis. On the other hand, ciglitazone, which does not possess the same reactive *α*,*β*-unsaturated carbonyl group as 15d-PGJ_2_, did not affect ROS and glutathione production, underscoring the differential actions of different PPAR*γ* ligands.

Recently, studies started to focus on the role of PPAR*γ* in B cell physiology. To investigate the intrinsic effects of PPAR*γ* itself (rather than ligand induced activation), PPAR*γ* expression was modulated using siRNA or a PPAR*γ*-expressing lentiviral vector in Ramos B lymphoma cell line [122]. In B cells with PPAR*γ* knockdown, cellular proliferation and NF-*κ*B translocation were enhanced upon stimulation. Interestingly, B cells were less differentiated, with increased expression of CD19 and CD20 and reduction in CD38. This was the first evidence showing that PPAR*γ* could be involved in regulating B cell differentiation. Similarly, PPAR*γ* overexpression led to reduced B cell proliferation and a more differentiated phenotype. Moreover, B cells upregulate PPAR*γ* expression upon stimulation, implying the regulatory role of PPAR*γ* during B cell activation [123].

PPAR*γ* ligands can also act to alter B cell differentiation and antibody production. 15d-PGJ_2_ enhanced antibody production, including IgM and IgG, and this was blunted with GW9662. While B cell proliferation or antibody production can contribute towards chronic inflammation, under certain contexts—like a viral infection or a vaccine response—enhanced antibody production may enhance viral clearance and speed a return to homeostasis. In addition to antibody production, the percentage of antibody-secreting cells (CD27+CD38+) and the expression of the transcription factor Blimp-1 (which is involved in B cell plasma cell differentiation) were also increased. These effects were mediated by Cox-2 and the addition of a Cox-2 selective inhibitor attenuated IgM and IgG production induced by 15d-PGJ_2_. The ability of PPAR*γ* ligands to enhance B cell differentiation, though, was only partially PPAR*γ*-dependent, as a PPAR*γ* inhibitor blocked enhanced IgG production but not IgM production, suggesting that PPAR*γ* might be involved in B cell class switching [123].

In our lab, the role of PPAR*γ* in B cells has further been investigated using B cell-specific PPAR*γ* knockout mice [124]. Even though some studies have shown the suppressive effects of PPAR*γ* ligands on primary mouse bone marrow B cell proliferation* in vitro* [125], no significant differences were observed in splenic and bone marrow B cell numbers in our studies. In addition, serum antibody levels in B cell-specific PPAR*γ*-deficient mice were similar to those in wild type mice. However, when PPAR*γ*-deficient mice were challenged with the ovalbumin antigen, primary immune responses were impaired. Serum antigen-specific antibody levels, percentage of germinal center B cells, and CD138+ plasma cells were significantly lower than wild type mice. Moreover, the memory response was also impaired against reinjection with the antigen. Other investigators have studied B cells derived from PPAR*γ* haploinsufficient (PPAR*γ* ^+/−^) mice with 50% reductions in PPAR*γ* [126]. These mice have enhanced B cell proliferation and increased serum IgM and IgG production, but since the PPAR*γ* knockout is not B cell specific, the loss of PPAR*γ* in other immune cells, such as T cells, may cause immune response changes that in turn alter B cell function. The anti-inflammatory effects of 15d-PGJ_2_ on B cell IgE production* in vitro* were also reported; in Burkitt's lymphoma cell line, DND39, IL-4-induced transcription of epsilon germline transcript was suppressed by 15d-PGJ_2_, in turn inhibiting B cell class switching to IgE [127]. These contributions of the adaptive immune system are equally important for resolution and homeostasis, and PPAR*γ* appears to work through B and T cells to mediate these effects.

## 9. PPAR***γ*** in Animal Models of Inflammation

Unchecked inflammation plays a role in the development and progression of many chronic diseases, with strong contributions from the innate immune system. PPAR*γ* remains an attractive therapeutic target for treatment of many diseases with underlying inflammatory pathology. Unlike other PPAR isoforms, PPAR*γ* knockout mice are embryonic, lethal [128]. Thus the majority of animal work investigating PPAR*γ* in animal models has utilized pharmacologic activation using synthetic or endogenous PPAR*γ* agonists, though conditional knockouts have been utilized to investigate tissue and cell type specific roles of PPAR*γ* [129, 130]. Much work has been done looking at the effects of PPAR in metabolism, insulin sensitivity, and obesity, which has led to the use of PPAR*γ* agonists as therapeutics for type 2 diabetes; these studies have been reviewed elsewhere. Here, we will highlight some of the other important inflammatory disease models in which PPAR*γ* plays a role.

### 9.1. Lung

PPAR*γ* is expressed in many cell types in the lung, leading to work investigating the potential for PPAR*γ* agonists as treatments in inflammatory lung diseases. In a mouse model of allergic asthma, ciglitazone treatment was able to reduce airway inflammation and mucus production [131]. A similar PPAR*γ*-dependent suppression of allergic inflammation was seen in a study using both pharmacologic activation of PPAR*γ* and viral gene transfer of PPAR*γ* cDNA [132]. These data support recent findings that asthmatics taking TZDs for treatment of diabetes had a reduced risk for asthma exacerbation [133]. Other nonallergic models of lung inflammation have highlighted the potential beneficial roles of PPAR*γ* ligands. Inflammation from the profibrotic drug bleomycin is reduced in mice treated with the synthetic agonist me-CDDO [38]. A recent study showed that the loss of epithelial PPAR*γ* in the lung enhanced inflammatory mediator production, immune cell recruitment, and exacerbating emphysematous changes following chronic smoke exposure [129]. Pharmacological activation in wild type mice with PPAR*γ* agonists further protected against inflammation following smoke exposure. Another report showed treatment with rosiglitazone, both prophylactically and therapeutically, reduced inflammatory cell counts in the bronchoalveolar lavage fluid in response to four weeks of cigarette exposure. However, inflammatory mediator levels were only reduced with prophylactic treatment, suggesting that the therapeutic effects of PPAR*γ* may be independent of changes in inflammatory mediators [134]. When an exacerbatory infection with nontypeable* Haemophilus influenzae* was added, rosiglitazone reduced neutrophil influx into the lung without compromising bacterial clearance, though no differences in the inflammatory signal IL-1*α*, MCP-1, or CXCL5 were detected in the BALF, further supporting the concept that PPAR*γ* therapeutic effects are not due to simply regulating inflammatory mediator production [134]. Interestingly, in a mouse model of influenza infection, treatment with 15d-PGJ_2_ starting one day after infection dampened inflammatory mediator gene transcription and increased mouse survival and decreased weight loss [135]. No protection was seen if 15d-PGJ_2_ treatment started on day 0. In conclusion, the ability of PPAR*γ* to regulate pulmonary inflammatory mediators seems to be important in protecting against future insult, whereas the mechanisms of PPAR*γ* mediated therapeutic benefits are less clear.

### 9.2. Colon

The role of PPAR*γ* in inflammation in other organ systems has also been investigated. Colonic tissue has high expression levels of PPAR*γ*. Indeed, synthetic [136, 137] and endogenous [130, 138] PPAR*γ* ligands have been shown to be efficacious in reducing inflammation in mouse and rat models of experimental colitis. Similar findings have been seen in a pig model of bacterial colitis, where conjugated linoleic acid (CLA) supplementation reduced inflammatory-induced mucosal damage [139]. Loss of function studies have shown that mice that are heterozygous deficient for PPAR*γ* are more susceptible to inflammation from intestinal ischemia/reperfusion injury [140] and colitis [141]. In support of this, tissue specific knockout of PPAR*γ* in the colon prevents the ability of CLA to protect against dextran sodium sulfate induced colitis [130]. Another study found that during colitis PPAR*γ* RNA levels are decreased in the intestinal lamina propria and peritoneal exudate and that adenoviral gene transfer of PPAR*γ* rescued sensitivity to PPAR*γ* ligands and reduced markers of inflammation and improved mouse survival [142].

### 9.3. Central Nervous System

Inflammation contributes to many neurodegenerative diseases, including Alzheimer's disease and Parkinson's disease, and indeed PPAR*γ* activation has shown beneficial effects in many animal models [143]. A study using a transgenic model of Alzheimer's disease showed that rosiglitazone treatment reduced the appearance of A*β* plaques in multiple areas of the brain [144]. This was accompanied by the reduction of the RNA levels of the proinflammatory markers Cox-2 and TNF*α*. In a model of Parkinson's disease, chronic treatment with rosiglitazone reduced microglial activation and neuronal loss which corresponded with improved behavioral function [145]. Similar results have been seen with pioglitazone and with a novel PPAR*γ* ligand, MDG548 [146–148]. PPAR*γ* has been tested in experimental models of experimental allergic encephalomyelitis (EAE) as model for multiple sclerosis. 15d-PGJ_2_ and ciglitazone treatment were able to reduce inflammation and demyelination in mice immunized with mouse spinal cord homogenate [149]. Similarly, troglitazone treatment reduced EAE lesion size and clinical score which correlated with decreased transcripts of TNF*α* and IL-1*β* [150].

PPAR*γ* has shown protective effects in many models of disease and shows both the ability to protect against inflammation and stimulate recovery. Although most work shows regulation of inflammation, the role of PPAR*γ* requires further study as beneficial actions can be seen even after inflammation has been established, suggesting therapeutic actions for PPAR*γ* in promoting resolution that may be independent of the regulation of proinflammatory mediators.

## 10. Specialized Proresolving Mediators: Emerging Players in Resolution

The consumption of dietary omega-3 polyunsaturated fatty acids (*ω*-3 PUFAs), such as eicosapentaenoic acid (EPA) and docosahexaenoic acid (DHA), confers numerous reported health benefits, including an improved prognosis in a wide variety of chronic inflammatory diseases [151]. While the mechanisms underlying these benefits remain largely unknown, EPA and DHA are natural PPAR*γ* ligands [152, 153], and a growing body of* in vitro* and* in vivo* evidence indicates that these *ω*-3 PUFAs exert at least some of their anti-inflammatory and proresolving effects via PPAR*γ* activation. Specialized proresolving mediators (SPMs) constitute a novel and growing array of endogenously produced, lipid-derived compounds that actively promote the resolution of inflammation; many SPMs are metabolites of *ω*-3 PUFAs, and an exciting biological circuitry connecting SPMs and PPAR*γ* is now beginning to emerge [1].

*ω*-3 PUFAs exert anti-inflammatory and proresolving effects in multiple organs; growing evidence shows the importance of PPAR*γ* in mediating these effects. For instance, in an immortalized human proximal renal tubular cell line (i.e., human kidney 2 (HK-2) cells), both EPA and DHA increase PPAR*γ* mRNA and protein expression and attenuate LPS-induced activation of NF-*κ*B and expression of monocyte chemoattractant protein-1. Importantly, treatment with the PPAR*γ* agonist/antagonist bisphenol A diglycidyl ether (BADGE) inhibits the activation of PPAR*γ* by EPA and DHA and abolishes both *ω*-3 PUFAs' inhibitory effects on LPS-induced NF-*κ*B activation in HK-2 cells [154].

Unsurprisingly, the anti-inflammatory and proresolving effects of *ω*-3 PUFAs are also mediated in part by influences on innate immune cells, and many of these influences appear to be PPAR*γ*-dependent. For instance, in human bone marrow derived dendritic cells (DCs), exposure to DHA inhibits expression of proinflammatory IL-12 via a mechanism that is in part dependent on activation of PPAR*γ* [155]. Similarly, in human monocyte-derived DCs, DHA diminishes both the expression of IL-12 and the capacity of DCs to activate autologous T cells; these effects are also abolished by cotreatment with GW9662, indicating PPAR*γ* dependence [156]. In murine macrophage-like RAW264.7 cells, DHA increases PPAR*γ* mRNA expression and nuclear translocation, enhances phagocytosis and efferocytosis, induces M2 polarization (as indicated by mRNA levels of CD36, IL-10, and TGF*β*; surface expression of CD36 protein; and secretion of IL-10 and TGF*β*), and inhibits LPS-induced M1 polarization (as indicated by production of proinflammatory cytokines TNF*α*, IL-1*β*, and IL-6). Knockdown of PPAR*γ* using siRNA abolishes the stimulatory effects of DHA on efferocytosis and M2 polarization [157].

Human clinical studies have begun to link *ω*-3 PUFAs and PPARs. In a recent study, patients consumed a moderately high dose of *ω*-3 PUFAs (3.4 g/day of EPA and DHA ethyl esters) for 2-3 weeks prior to undergoing elective cardiac surgery. Compared to that of untreated controls, the atrial myocardium of patients given *ω*-3 PUFA had greater transactivation of PPAR*γ* and higher mRNA levels of several genes known to be activated by PPAR*γ*, including CD36, heart-type fatty acid binding protein, and long-chain acyl-CoA dehydrogenase; furthermore, atrial tissue from *ω*-3 PUFA-treated patients had enhanced mitochondrial respiration supported by palmitoyl-carnitine [158]. More work remains to elucidate the extent to which PPAR*γ* activation mediates *ω*-3 PUFA-induced enhancement of mitochondrial fatty acid oxidation and antioxidant capacity in human atrial myocardium; yet, this translational research underscores the growing recognition of the link between *ω*-3 PUFAs and PPAR*γ* activation.

Studies demonstrating PPAR*γ*-dependent anti-inflammatory effects of *ω*-3 PUFAs inspire the hypothesis that active EPA or DHA metabolites, including SPMs, may act as PPAR*γ* ligands to exert anti-inflammatory effects. Because (1) SPMs and PPAR*γ* ligands share many overlapping anti-inflammatory and proresolving functions, (2) many SPMs are derived from known PPAR*γ* ligands such as EPA and DHA, and (3) PPARs have wide binding sites that can accommodate a variety of ligands, several researchers have postulated that SPMs could act as direct PPAR ligands. Krishnamoorthy and colleagues used cell-based luciferase reporters to test ability of RvD1 and RvE1 to activate PPAR*α*, PPAR*δ*, PPAR*γ*, and RXR*α*; neither RvD1 nor RvE1 induced strong activation of any of the PPARs and RXRs tested. [159]. On the other hand, there is mounting evidence that some SPMs may act as PPAR*γ* activators and that some of the effects of SPMs are at least in part PPAR dependent. For example, one group administered LPS intratracheally to BALB/c mice to model acute lung injury (ALI). Administration of RvD1 intravenously prior to LPS exposure significantly abrogated LPS-induced inflammation, as indicated by histologic ALI score, and blunted elevations in BALF neutrophils, TNF*α*, and IL-6; RvD1 treatment also significantly inhibited LPS-induced I*κ*B*α* degradation, NF-*κ*B p65 subunit nuclear translocation, and DNA-binding activity of NF-*κ*B. Furthermore, RvD1 increased protein levels of PPAR*γ* in the nucleus of lung tissues. Importantly, all of these effects of RvD1 were partially reversed by pretreating mice intravenously with the PPAR*γ* antagonist GW9662, suggesting that RvD1 attenuates LPS-induced ALI via a mechanism that is at least somewhat PPAR*γ*-dependent [160].

Of the SPMs studied to date, protectins have shown the strongest associations with PPAR*γ*. Using an adipogenesis assay and a PPAR*γ* transactivation reporter, Bazan and colleagues demonstrated that neuroprotection D1 (NPD1) is a direct PPAR*γ* activator. Furthermore, the group reported PPAR*γ*-dependent effects of NPD1 in* in vitro* and 3x-Tg-AD mouse models of Alzheimer's disease. Decreases in hippocampal levels of neuroprotective DHA and NPD1 were detected by mass spectrometry in older wild type and Alzheimer's mice. NPD1 repressed proamyloidogenic processing of *β*-amyloid polypeptide via the *β*-secretase pathway and enhanced nonamyloidogenic processing via *α*-secretase in primary human neuronal glia. In contrast, rosiglitazone treatment or transient PPAR*γ* overexpression only inhibited the *β*-secretase pathway with no effects on the *α*-secretase pathway. Accordingly, the PPAR*γ* antagonist GW9662 abrogated NPD1's modulation of the *β*-secretase pathway but not its stimulatory effect on the *α*-secretase pathway. These results indicate that NPD1's antiamyloidogenic effects are partly dependent on its ability to activate PPAR*γ* [161].

Additional evidence for PPAR*γ* activation by protectins came from Marette and colleagues, who studied the visceral adipose tissue of* fat-1* transgenic mice. These mice convert endogenous *ω*-6 to *ω*-3 PUFA; when fed a high-fat diet (HFD) rich in *ω*-6 PUFA,* fat-1* mice maintain an adipose tissue *ω*-3 : *ω*-6 ratio of 1 : 1, compared to 50 : 1 in wild type counterparts. A microarray of epididymal adipose tissue revealed upregulation of PPAR*γ* and RXR*γ* in HFD-fed* fat-1* mice. Additionally, compared with HFD-fed wild type mice, HFD-fed* fat-1* mice synthesized much higher epididymal adipose tissue levels of protectin DX (PDX), which along with PD1 was identified as a PPAR*γ* agonist [162].

Another set of experiments suggests not only that some SPMs can activate PPAR*γ*, but also that some* in vivo* actions of PPAR*γ* agonists may be mediated by effects on SPM biosynthesis. In addition to its proresolving effects described above, rosiglitazone induced the expression of 5-lipoxygenase (5-LO), the dioxygenase that (a) catalyzes the conversion of arachidonic acid to 5-HPETE, the precursor to the proinflammatory leukotrienes, and (b) catalyzes the biosynthesis of proresolving lipoxins, in a rodent model of stroke using middle cerebral artery occlusion (MCAO) [163]. Notably, pharmacologic inhibition of 5-LO using BWA4C dose-dependently diminished the neuroprotective and anti-inflammatory effects of rosiglitazone in the ischemic brain. Moreover, rosiglitazone amplified PPAR*γ* transcriptional activity and induced the synthesis of LxA4 in the ischemic cortex of MCAO-treated rats; both of these effects were abolished by the 5-LO inhibitor BWA4C. Based on this finding, the authors administered LxA4 intracerebroventricularly to rats undergoing MCAO and noted neuroprotection as indicated by reduced infarct volume and improved neurological deficit scores; surprisingly, administration of the PPAR*γ* antagonist T0070907 reversed the beneficial effects of LxA4, suggesting its effects are partially PPAR*γ*-dependent. Indeed, in isolated nuclei from rat cerebral cortex, incubation with LxA4 increased PPAR*γ* transcriptional activity [163].

Taken together, these studies demonstrate a complex link between SPMs and PPAR*γ* and suggest the existence of feed-forward amplification loops wherein PPAR*γ* agonists activate PPAR*γ*, which in turn activates biosynthetic pathways that produce SPMs. This increase in SPMs can further augment PPAR*γ* activation in order to mediate anti-inflammatory, proresolving effects. Much more work remains to better understand the implications of context-dependent crosstalk between *ω*-3 PUFAs, SPMs, and PPAR*γ* in health, disease, and the development of safe, effective, novel therapeutics.

## 11. Conclusions and Remaining Questions: PPAR***γ*** and the Resolution of Inflammation

**Figure 5:**
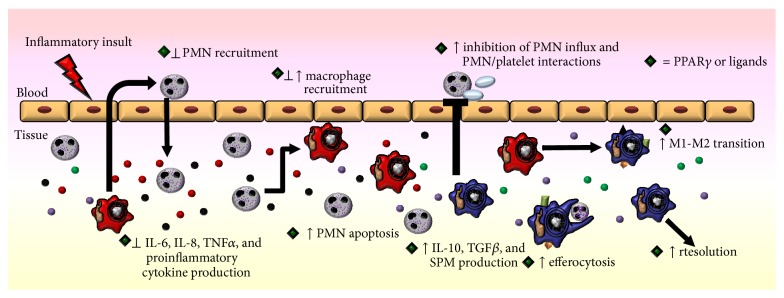
PPAR*γ* and the resolution of inflammation. PPAR*γ* and PPAR*γ* ligands (denoted by green diamond) play roles in all stages of inflammation. Early on, PPAR*γ* and its ligands decrease neutrophil recruitment and proinflammatory cytokine production. PPAR*γ* and its ligands then act to promote neutrophil apoptosis and efferocytosis and induction from proinflammatory to anti-inflammatory production. Finally, macrophages move to an M2 phenotype and tissue repair is initiated to return to homeostasis.

It is clear that PPAR*γ* plays critical roles in all phases of inflammation (Figure 5). This evidence is largely drawn from a broad evaluation of PPAR*γ* literature, but some studies comprehensively evaluated the role of PPAR*γ* throughout the inflammatory and resolving phases. In a model of murine chronic granulomatous disease pioglitazone increased TGF*β* and IL-10 while decreasing KC (mouse homologue to IL-8), IL-6, and TNF*α* expression. Pioglitazone treated mice had a decrease in the total number of neutrophils and enhanced efferocytosis of apoptotic neutrophils. This clearance was mediated by macrophages, which had increased PPAR*γ* and CD36 expression following treatment. Importantly, these results were seen when pioglitazone was administered as a pretreatment and when pioglitazone was given after the onset of inflammation, indicating that it is truly during the resolution phase of inflammation that PPAR*γ* is playing a role [164].

Several important questions remain regarding PPAR*γ*'s role in modulating inflammation. First and foremost, the dependent versus independent effects of PPAR*γ* ligands need further elucidation. Many studies have made use of a wide variety of techniques to block PPAR*γ* signaling, including PPAR*γ* knockout or knockdown, antagonists, and siRNAs; these methods, particularly antagonists, can have off-target effects which should be closely evaluated. Other studies have evaluated induction of PPAR*γ* expression or PPAR*γ*-dependent luciferase reporters, but few studies show direct PPAR*γ* binding. Furthermore, many studies have used putative PPAR*γ* ligands without evaluating the dependency on PPAR*γ* at all. All work using these ligands should more carefully evaluate the specificity of these effects, as more complete knowledge of PPAR*γ* ligand binding and activation is critical for therapeutic use.

Additionally, there is a lack of evidence regarding the temporal aspects of PPAR*γ* regulation. Most studies conducted involved a pretreatment or single postexposure dose of PPAR*γ* ligands, but given the variable effects between ligands (i.e., TZDs versus prostaglandins in enhancing or decreasing macrophage phagocytosis), these activators may have the most beneficial effects when administered at different phases of the inflammatory and resolving processes. Overall, the emerging literature regarding the proresolving roles of PPAR*γ*, particularly in the context of innate immunity, is encouraging and opens up new questions for investigation and new opportunities for therapeutic use of PPAR*γ* ligands.

## Figures and Tables

**Table 1:** List of PPAR*γ* ligands.

Prostaglandins	Thiazolidinediones	Eicosanoids	Other lipids
Prostaglandin D_2_^*∗*^	Rosiglitazone^+^	Arachidonic acid^*∗*^	Conjugated linoleic acids^*∗*^
15-Deoxy prostaglandin J_2_^*∗*^	Pioglitazone^+^	SPMs^*∗*^	Oxidized LDLs^+^
Prostaglandin A1^*∗*^	Ciglitazone^+^	15-HETE^*∗*^	
	Troglitazone^+^	DHA^*∗*^	
		EPA^*∗*^	

Fibrates	Nutraceuticals	NSAIDs	Triterpenoids

Clofibrate^+^	Genistein^*∗*^	Indomethacin^+^	Oleanolic acid^*∗*^
Fenofibrate^+^	Biochanin A^*∗*^	Ibuprofen^+^	CDDO^+^
Gemfibrozil^+^	Daidzein^*∗*^		
Ciprofibrate^+^	Hesperidin^*∗*^		

^*∗*^Synthetic ligand, ^+^endogenous ligand.

SPMs: specialized proresolving mediators; HETE: hydroxyeicosatetraenoic acid; DHA: docosahexaenoic acid; EPA: eicosapentaenoic acid; LDLs: low density lipoproteins; CDDO: 2-cyano-3,12-dioxo-oleana-1,9(11)-dien-28-oic acid; NSAIDs: nonsteroidal anti-inflammatory drugs.

## Abbreviations

15d-PGJ_2_: 15-Deoxy prostaglandin J_2_
ALI: Acute lung injury
BADGE: Bisphenol A diglycidyl ether
Cox-2: Cyclooxygenase-2
CRP: C-reactive protein
DHA: Docosahexaenoic acid
EPA: Eicosapentaenoic acid
HFD: High-fat diet
LDLs: Low density lipoproteins
CDDO: 2-Cyano-3,12-dioxo-oleana-1,9(11)-dien-28-oic acid
CLA: Conjugated linoleic acid
IL: Interleukin
LPS: Lipopolysaccharide
MCAO: Middle cerebral artery occlusion
MCP-1: Monocyte chemoattractant protein-1
MPO: Myeloperoxidase
NPD1: Neuroprotectin D1
OA: Oleanolic acid
*ω*-3 PUFAs: Omega-3 polyunsaturated fatty acids
PAI-1: Plasminogen activator inhibitor 1
PDX: Protectin DX
PPARs: Peroxisome proliferator activated receptors
PGD_2_: Prostaglandin D_2_
RXR*α*: Retinoid X receptor alpha
SPMs: Specialized proresolving mediators
TNF*α*: Tumor necrosis factor-alpha
TZDs: Thiazolidinediones.
